# Generation of multi-layered protein bodies in *N. benthamiana* for the encapsulation of vaccine antigens

**DOI:** 10.3389/fpls.2023.1109270

**Published:** 2023-01-17

**Authors:** Jennifer Schwestka, Lukas Zeh, Marc Tschofen, Fabian Schubert, Elsa Arcalis, Maria Esteve-Gasent, Emanuela Pedrazzini, Alessandro Vitale, Eva Stoger

**Affiliations:** ^1^ Institute of Plant Biotechnology and Cell Biology, Department of Applied Genetics and Cell Biology, University of Natural Resources and Life Sciences, Vienna, Austria; ^2^ Department of Veterinary Pathobiology, College of Veterinary Medicine, College Station, TX, United States; ^3^ Istituto di Biologia e Biotecnologia Agraria, Consiglio Nazionale delle Ricerche (CNR), Milano, Italy

**Keywords:** plant-based vaccines, storage organelles, prolamin bodies, microparticles, bioencapsulation, vaccine delivery

## Abstract

The ability of plants to assemble particulate structures such as virus-like particles and protein storage organelles allows the direct bioencapsulation of recombinant proteins during the manufacturing process, which holds promise for the development of new drug delivery vehicles. Storage organelles found in plants such as protein bodies (PBs) have been successfully used as tools for accumulation and encapsulation of recombinant proteins. The fusion of sequences derived from 27-kDa-γ-zein, a major storage protein of maize, with a protein of interest leads to the incorporation of the chimeric protein into the stable and protected environment inside newly induced PBs. While this procedure has proven successful for several, but not all recombinant proteins, the aim of this study was to refine the technology by using a combination of PB-forming proteins, thereby generating multi-layered protein assemblies in *N. benthamiana*. We used fluorescent proteins to demonstrate that up to three proteinaceous components can be incorporated into different layers. In addition to 27-kDa-γ-zein, which is essential for PB initiation, 16-kDa-γ-zein was identified as a key element to promote the incorporation of a third zein-component into the core of the PBs. We show that a vaccine antigen could be incorporated into the matrix of multi-layered PBs, and the protein microparticles were characterized by confocal and electron microscopy as well as flow cytometry. In future, this approach will enable the generation of designer PBs that serve as drug carriers and integrate multiple components that can be functionalized in different ways.

## 1 Introduction

The diversity of drugs, particularly biologics, with different mechanisms of action presents unique challenges for drug delivery and has led to the development of new delivery systems that facilitate transport across biological barriers, delay degradation, or alter the distribution of a drug to increase its efficacy and/or reduce its toxicity (Boyd, 2008). For example, incorporation of drugs into liposomes, nanoparticles, microparticles, or polymer-based carriers may protect the cargo from environmental effects and enable controlled and targeted delivery of the active ingredients (Schwestka and Stoger, 2021). Particle-based delivery systems also offer opportunities for increasing the efficacy of vaccines by enhancing their stability and ability to interact with target cells *in vivo* (Scotti and Rybicki, 2013; Marsian and Lomonossoff, 2016; Schwestka and Stoger, 2021; Ward et al., 2021). Particulate structures also possess inherent immunostimulatory properties, promoting more efficient uptake by antigen presenting cells than corresponding soluble antigens (Zhu et al., 2014; Snapper, 2018). Particulate protein assemblies display multiple copies of the antigen, typically with regular spacing, which enhances the immune response, and the particle itself may also confer adjuvant properties (Zhu et al., 2014; Jiao et al., 2014; Snapper, 2018; Torres et al., 2019).

Encapsulation is particularly useful for the mucosal delivery of vaccines, which induces not only systemic immunity but also protects mucosal barriers such as the intestine and respiratory tract from invading pathogens (Zhu and Berzofsky, 2013; Kim and Jang, 2017). Such non-invasive drug delivery strategies are particularly suitable for the administration of veterinary vaccines to control diseases that pose an economic risk for farmers and/or the risk of zoonotic transmission to humans. Mucosal immunization, such as the delivery of vaccines *via* drinking water or feed enables the vaccination of large numbers of animals over a short period of time without the effort and stress caused by injections. Most currently licensed veterinary mucosal vaccines are based on live-attenuated viruses and bacteria, but due to the risks associated with those vaccines, there is interest for new vaccine technologies involving synthetic particle carriers to combat emerging zoonotic diseases (Vacher et al., 2013; Topp et al., 2016; Mohan et al., 2017; Schwestka et al., 2020).

One way to achieve encapsulation of pharmaceutical compounds is based on *in vitro* techniques such as coacervation and spray drying (Perry and McClements, 2020). In this context, natural plant-derived polymers such as storage proteins, cell-wall carbohydrates and starch are often extracted and reformulated to encapsulate drugs *in vitro*. For example, certain subfractions of zeins, the large family of maize storage proteins, have been studied due to their unique physicochemical and biological properties. They form edible films that are tough, hydrophobic and resistant to microbial degradation (Shukla and Cheryan, 2001), allowing the use of zein nanoparticles for the *in vitro* encapsulation of pharmaceutical and nutraceutical compounds (Patel et al., 2010; Luo et al., 2011; Karthikeyan et al., 2014; Penalva et al., 2017). Given the ability of plants to assemble protein storage organelles, which are particulate structures formed in the endoplasmic reticulum (ER) or vacuoles, mainly consisting of seed storage proteins such as zeins, recombinant proteins can also be directly bioencapsulated during the manufacturing process when plants are used as production hosts. In addition, this strategy can leverage many other advantages that plants offer in providing a safe, scalable, and cost-effective platform for the rapid and sustainable production of vaccines and drugs (Lico et al., 2020; Capell et al., 2020; Margolin et al., 2020; Rosales-Mendoza, 2020). Natural plant storage organelles are mostly restricted to seeds and tubers, and these organs have been used to achieve the *in vivo* encapsulation of proteins by directing them to accumulate in storage organelles holding promise for the development of new drug delivery vehicles (Stoger et al., 2005; Takaiwa et al., 2015; Endo et al., 2021). For example, transgenic rice seeds have been used for the production and delivery of T-cell epitopes representing allergenic proteins (Takagi et al., 2010; Takaiwa et al., 2015; Ishida et al., 2021). By targeting cedar pollen allergens to protein storage organelles such as ER-derived protein bodies (PBs) and protein storage vacuoles, the encapsulated allergens were protected against proteolytic digestion and long-term oral administration of transgenic rice improved medication scores long-term administration (Takaiwa et al., 2019; Endo et al., 2021). While this is a successful approach and cereal seeds are valuable bioreactors for the production and storage of high-value compounds (Zhu et al., 2022), the generation of transgenic cereals is a lengthy process, rendering seed-based systems most suitable for products that are required in large quantities and over long periods of time (Tschofen et al., 2016; Vamvaka et al., 2016). Vaccines are often needed on a seasonal basis and have to be adapted to novel pathogens favoring transient expression systems involving the short-term expression of proteins in plants infiltrated with recombinant bacteria. Such systems are widely used, and several large-scale production facilities have been established in the last 10 years based on the tobacco relative *Nicotiana benthamiana* (Lomonossoff and D’Aoust, 2016; Fischer and Buyel, 2020; Kurokawa et al., 2021). Although the process of PB biogenesis is not fully understood, some aspects have been characterized in sufficient detail to induce these protein storage organelles ectopically in non-storage tissues such as leaves of *Nicotiana benthamiana*, and this offers a faster, more versatile and controllable method to encapsulate proteins in the protective environment of PBs. Usually this has been achieved by means of fusion proteins joining the therapeutic candidate to a partial prolamin sequence. For example, fusion to the N-terminal part of 27-kDa-γ-zein (also called Zera) can induce the formation of ectopic PBs in heterologous expression systems (Mainieri et al., 2004; De Virgilio et al., 2008; Torrent et al., 2009b). The resulting PBs are spherical particles with unique properties (~1 µm diameter, ~1.20 g/cm³ density) that allow separation during downstream processing, which can be either based on gradient ultracentrifugation (Whitehead et al., 2014; Van Zyl et al., 2017) or serial tangential flow filtration steps (Schwestka et al., 2020).

Several recombinant pharmaceutical proteins have been produced in an active form by inducing ectopic PBs in *N. benthamiana* leaves, including calcitonin, human epidermal growth factor and growth hormones, as well as vaccine candidates (De Virgilio et al., 2008; Torrent et al., 2009a; Whitehead et al., 2014; Hofbauer et al., 2016). In addition to incorporating and protecting recombinant proteins zein–antigen microparticles were found to function as adjuvants, triggering a stronger immune response than the soluble antigen. This was shown in mice where the immune response to antigens incorporated into PBs could not be further enhanced by adding adjuvant (Hofbauer et al., 2016). Empty PBs administered together with the soluble antigen also enhance the immune response (Whitehead et al., 2014). Another important characteristic of γ-zein PBs is their ability to interact with cell membranes, likely reflecting the presence of proline-rich repeats in the 27-kDa-γ-zein polypeptide, which share some similarity with cell-penetrating peptides (Kogan et al., 2001; Fernandez-Carneado et al., 2004). We recently showed that γ-zein-EGFP (enhanced green fluorescent protein) PBs were taken up more efficiently than synthetic polystyrene particles of the same size when administered to intestinal epithelial cells and to antigen-presenting cells (Schwestka et al., 2020).

However, the induction of artificial PBs is not always successful when the Zera sequence is used as a fusion tag. For some recombinant viral antigens, including the HIV negative factor (Nef) and CAP256 gp140 envelope, the addition of Zera was not sufficient to lead to PB formation, although the fusion antigen accumulated in the ER (De Virgilio et al., 2008; Ceresoli et al., 2016; Ximba et al., 2020).

We therefore embarked on a novel synthetic approach aiming at the *in vivo* generation of designer PBs with a core-shell structure that may be applicable to a wider range of protein pharmaceuticals and allow the incorporation of several components and functions into the same PB. Based on existing knowledge on the mechanisms of storage organelle formation and on the interaction between protein components of naturally occurring multi-layered PBs found in maize seeds (Lending and Larkins, 1989) we explored selected members of the zein protein family in a combinatorial approach. Natural PBs of maize are composed of a shell containing γ- and β-zeins, and a core containing α-and δ-zeins. The γ- and β-zeins are expressed early in seed maturation, and are rich in cysteines and cross-linked by disulfide bonds. After PB initiation by 27-kDa-γ-zein, the remaining γ-zeins as well as 15-kDa-β-zein constitute the early PB, while α-and δ-zeins are only later incorporated by penetrating the existing matrix, which requires the rearrangement of existing disulfide bonds (Lending and Larkins, 1989; Guo et al., 2013). The 19-kDa-α-zein (B1) is the most abundant zein in the core of native zein PBs in maize, and δ-zeins are interesting candidates for the generation of layered PBs as well because they colocalize in the core of maize PBs alongside α-zein (Bagga et al., 1997; Guo et al., 2013; Kim and Krishnan, 2019).

We therefore included in our study 27-kDa-γ-zein, Zera, 16-kDa-γ-zein, 15-kDa-β-zein, 22-kDa-α-zein, 19-kDa-α-zein (B1) and 10-kDa-δ-zein. Each of these zeins was fused to at least two different fluorescent protein tags to allow for different color combinations upon co-expression in *N. benthamiana*. We selected the best combination for obtaining multi-layered PBs and tested it for PB formation with an antigen peptide derived from the outer surface protein BB0172 of *B. burgdorferi*, that was earlier identified as suitable candidate for the development of a veterinary vaccine for protection against Lyme disease (Small et al., 2014).

## 2 Material and methods

### 2.1 Molecular cloning

Constructs 19-kDa-α-zein-mCherry and Zera-EGFP were delivered in a pBluescriptIISK+ background and transferred into the binary pTRA vector (Maclean et al., 2007) by SmiI/XbaI-mediated restriction cloning. Synthesis by GeneCust Europe provided following constructs in the pTRA vector: 22-kDa-α-zein-mCitrine-Flag, 16-kDa-γ-zein-mKO2-cMyc, 15-kDa-β-zein-mTagBFP2-VSV, 10-kDa-δ-zein-V5, 19-kDa-α-zein-mCherry-HA, and Zera-Opt3a12-Flag. Recombination of BamHI/XbaI-digested DNA fragments generated the final constructs Zera-mTagBFP2- VSV, 19-kDa-α-zein-EGFP, 22-kDa-α-zein-EGFP, 22-kDa-α-zein-mCherry-HA, 22-kDa-α-zein-mTagBFP2-VSV, 16-kDa-γ-zein-EGFP, 16-kDa-γ-zein-mCherry-HA, 16-kDa-γ-zein-mTagBFP2-VSV, 15-kDa-β-zein-EGFP, 15-kDa-β-zein-mCherry-HA, 10-kDa-δ-zein-EGFP, 10-kDa-δ-zein-mCherry-HA, and 15-kDa-β-zein-Opt3a12-Flag. All zeins include their native signal peptide and neither zeins nor fluorescent proteins were codon optimized for the expression in *Nicotiana benthamiana*. All constructs were designed with a (GGGGS)2 linker between zein and the fluorescent protein. Constructs that contain a C-terminal peptide tag have their tag attached by a G3 linker after the fluorescent protein. Constructs that do not contain native zein UTRs instead harbor a UTR from tobacco etch virus (TEV) for increased mRNA stability. The pTRA vector is a derivative of pPAM (GenBank accession number AY027531) and contains the 35S promoter with duplicated transcriptional enhancer and the 35S terminator both originating from Cauliflower mosaic virus (CaMV) as well as matrix attachment regions of tobacco Rb7 up- and downstream of the promoter and terminator, respectively (Maclean et al., 2007; Sack et al., 2007). GenBank accession numbers for each zein are 19-kDa-α-zein (AF371269), 27-kDa-γ-zein (AF371261), Zera (N-terminal 93 amino acids of AF371261), 16-kDa-γ-zein (AF371262), 15-kDa-β-zein (AF371264), 10-kDa-δ-zein (AF371266), and 22-kDa-α-zein (AF371274). Opt3a12 consists of 12 repeats of a derivative of peptide B originating from the Borrelial outer membrane protein BB0172 (Small et al., 2014; Hassan et al., 2019).

### 2.2 Biological material

*Nicotiana benthamiana* plants were grown on soil in a chamber with a 16 h photoperiod at 70% relative humidity and day/night temperatures of 26°C and 16°C, respectively. pTRA constructs were transferred into chemically competent *Agrobacterium tumefaciens* GV3101 containing the helper plasmid pMP90RK (Koncz and Schell, 1986). Cultures of Agrobacterium strains were inoculated from glycerol cryo-stocks and cultivated in YEB medium containing 25 mg/l kanamycin, 25 mg/l rifampicin, and 50 mg/l carbenicillin. Cultures were incubated at 28°C while shaking at 200 rpm. Prior to infiltration, the cultures were pelleted and washed twice with infiltration medium (10 mM MES pH 5.6, 10 mM MgCl_2_, 100 µM acetosyringone). When Agrobacterium strains were combined for infiltration, equal amounts of agrobacteria were mixed and the final OD_600_ of the bacterial suspension was always adjusted to 0.3. Infiltration of *Nicotiana benthamiana* leaves was performed manually with 1-ml syringes, and leaves were harvested 7 days post infiltration (dpi).

### 2.3 Processing of plant material

Extraction and isolation of PBs was performed in a similar way as described previously (Schwestka et al., 2020). Since only small amounts of PBs were needed, small-scale purifications from 5-8 g leaf fresh weight (FW) were performed. The plant material was ground with mortar and pestle, precooled with liquid nitrogen, and then four volumes of extraction buffer (phosphate-buffered saline (PBS) + 2% Triton X-100) were added. The slurry was further homogenized with a disperser (IKA S 25 N - 10 G) and sonicated three times for 10 pulses (Branson Sonifier 450, VWR; power 2, duty cycle 50%) and incubated on ice for 15 minutes with agitation. The homogenate was further filtered through Miracloth to remove plant debris and the insoluble PBs were pelleted *via* centrifugation (30 minutes at 15,000 g and 4°C). The pellet was subsequently washed twice with extraction buffer and twice with PBS to remove the detergent. Afterwards a dead-end filtration with a series of filters of decreasing cut-offs (180, 120, 60, 30 and 10 μm, Merck Millipore Ltd., Nylon) was carried out and the filtrate was concentrated by centrifugation resuspended in a lower volume of PBS. This concentrate was then sonicated and subsequently applied onto a cushion of CsCl with a density of 1.45 g/cm^3^ to separate PBs (ρ = 1.29 g/cm^3^) from starch granules (ρ = 1.5 g/cm^3^). Centrifugation was carried out at 10,000 g for 30 minutes and the PBs were collected from the interface between the cushion and the applied sample. Ten volumes of PBS were added to the isolated PBs and the sample was centrifuged again, followed by two washing steps with PBS to remove residual CsCl.

### 2.4 Flow cytometry

Processed samples were measured in a V-bottom 96-well plate and data were collected for 10,000 events using a flow cytometer (CytoFlex S; Beckman Coulter). GFP and mCherry signal was excited at 488 nm and 561 nm, respectively and emission was measured at 525 and 610 nm, respectively. Forward, side scatter, GFP and mCherry gain was set to 40, 24, 50 and 300, respectively. Three independent measurements were performed from different agroinfiltrations. Gates for GFP-positive and mCherry-positive PBs were defined by using isolated Zera-GFP and Zera-mCherry PBs. Flow cytometry data were analyzed with CytExpert 2.4. (Beckman Coulter). An unpaired t-test was performed to test for significant differences (statistical difference was defined as p < 0.05) using GraphPad Prism 9 (9.3.1.).

### 2.5 Microscopy

#### 2.5.1 Confocal laser scanning microscopy

The expression and co-localization of the different proteins into PBs was analyzed with a Leica SP8 confocal microscope using a 63x water immersion lens with a numerical aperture of 1.2 and a refraction index of 1.33. Small pieces of infiltrated leaves (approximately 5x5 mm) were mounted in a drop of tap water on a microscopy slide. The power of the white light laser was set at 70%, the individual laser lines as well as the gain were adjusted according to requirements. Pictures were taken with a pixel density between 512x512 and 1024x1024 with line averaging between none and three. High line averaging or even frame averaging was not possible due to the high movement of the PBs. Pictures were taken with excitation at 405 nm (BFP), 485 nm (GFP) or 585 nm (mCherry) and emission at 422-448 nm, 502-529 nm or 599-635 nm, respectively. Images were acquired in sequential mode to avoid crosstalk between the channels of the individual fluorophores. Intensity profiles were recorded individually for each channel using Leica Application Suite X (3.5.7). Images from a minimum of three biological replicates (agroinfiltrations) were analyzed for each combination. In addition to the co-localization of the fluorophore fused zeins in individual PBs, the uniformity of the structures arising throughout a cell as well as throughout the whole tissue was analyzed. Pictures of individual PBs were taken in different focal planes and z-stacks with a step size of 100 nm.

#### 2.5.2 Fixation and embedding of leaf material for immunocytochemistry

Infiltrated leaves were cut into small pieces with a razor blade and fixed in 4% (w/v) paraformaldehyde plus 0.2% (v/v) glutaraldehyde in 0.1 M phosphate buffer (pH 7.4) at 4°C overnight. Samples were dehydrated through an ethanol series and polymerized in LR White resin as previously described (Arcalis et al., 2010). Ultrathin sections showing silver interferences were collected on copper grids and observed in a Tecnai G2 transmission electron microscope operating at 160 kV.

#### 2.5.3 Immunolocalization of embedded samples

Sections were blocked with 5% (w/v) bovine serum albumin in 0.1M phosphate buffer (pH 7.4) and incubated with a mix of primary antibodies (1:100, Mouse-anti-Flag, Goat-anti-GFP and Rabbit-anti-VSV) for 2 hours at room temperature.

After washing the sections with PBS containing 0.05% Tween-20, samples were incubated with donkey-anti-mouse, donkey-anti-goat and donkey-anti-rabbit-IgG linked to 6-nm, 18-nm and 10-nm colloidal gold, respectively.

### 2.6 Protein immunoblot analysis

Infiltrated leaf material was frozen in liquid nitrogen and ground with 3-mm metal beads using a swing mill and subsequently four volumes of extraction buffer (PBS + 2% Triton X-100) were added and incubated for 15 minutes on ice. After centrifugation for 5 min at 4°C, 14,000 g the supernatant was collected and concentrated reducing loading buffer was added. The pellet was re-extracted twice for 5 min with the same buffer to remove remaining soluble components. Subsequently the residual proteins were extracted from the pellet with the same volume of buffer containing 2% (w/v) SDS, 10% (w/v) glycerol, 50 mM DTT, 0.01% (w/v) Bromophenol blue, 60 mM Tris-HCl pH 6.8, 5 mM TCEP and 1M/7M Urea/Thiourea. Equal amounts of extract were incubated for 1 hour at 37°C to reduce and solubilize proteins in the pellet fraction. Samples were separated by sodium dodecylsulfate polyacrylamide gel electrophoresis (SDS-PAGE) on 12% (w/v) gels and transferred to a nitrocellulose membrane. The membrane was blocked with 5% (w/v) skimmed milk for 1 hour at room temperature and Flag-tagged proteins were detected using a mouse-anti-flag-tag antibody (Sigma, F3165; 1:10,000) and an alkaline phosphatase-conjugated donkey-anti-mouse IgG antibody (Promega; 1:10,000). For quantitative estimates, the samples were compared to serial dilutions of a flag-tagged standard protein (multiple tag, Genscript, M0101, 200, 500 and 1500 ng loaded on the gel) using Image Lab v5.1 (Bio-Rad Laboratories, Hercules, CA, USA). Estimates were based on three blots using three independent protein extracts of infiltrated leaves.

## 3 Results

### 3.1 Ectopic generation of double-layered protein bodies in *N. benthamiana*

Based on the known interactions between zeins and on their relative abundance within native PBs, we included in our study 27-kDa-γ-zein, Zera, 16-kDa-γ-zein, 15-kDa-β-zein, 22-kDa-α-zein, 19-kDa-α-zein (B1) and 10-kDa-δ-zein. Each of these zeins was fused to at least two different fluorescent protein tags and subcloned into identical expression cassettes (**Figures S1A, B**) to allow for different color combinations upon co-expression and to reveal any distorting effects caused by one specific fusion protein only or by an incidental interaction between two fusion tags.

**Figure 1:**
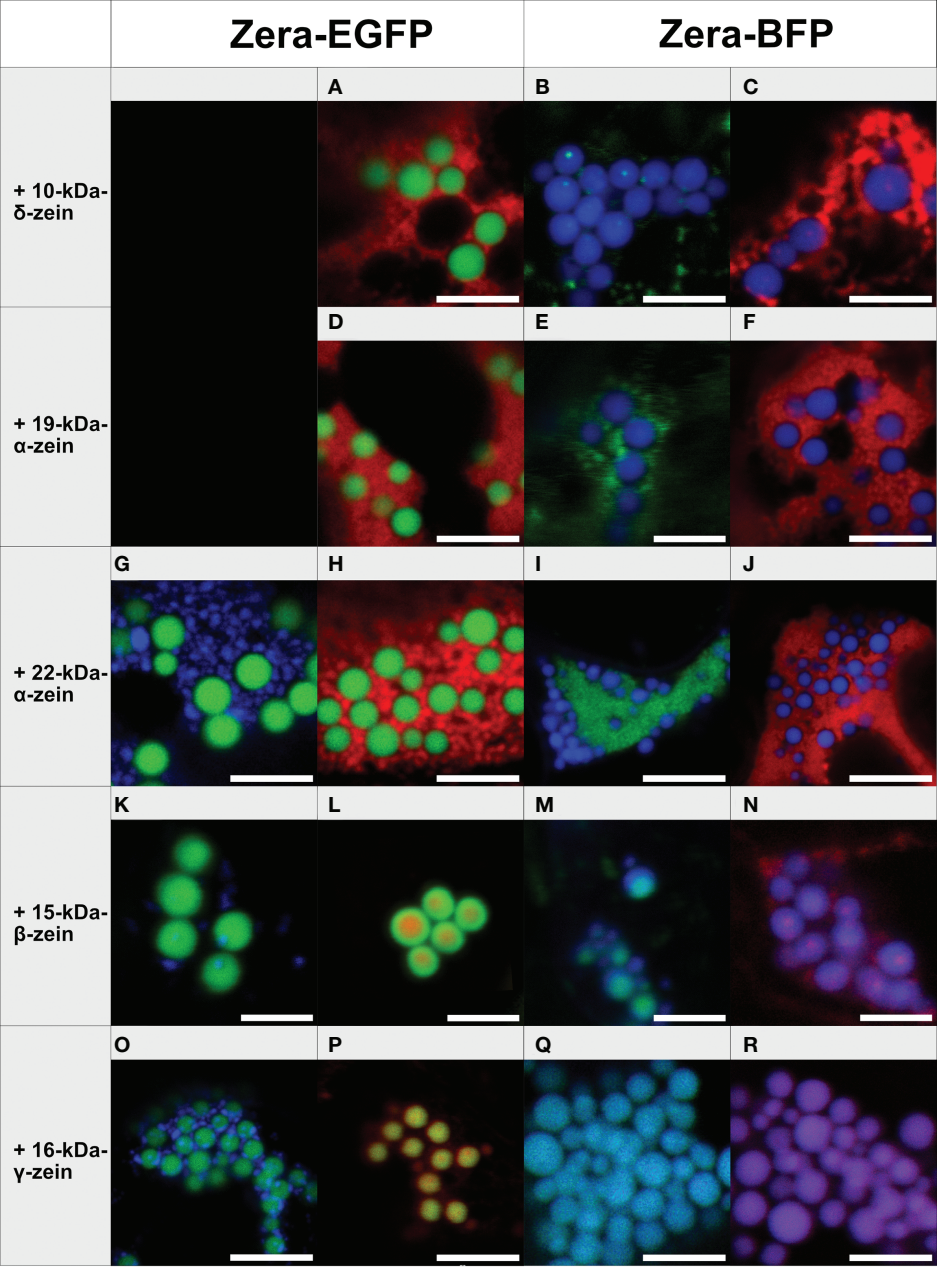
Co-expression of individual zeins with Zera-XFP in different fluorophore combinations (representative images recorded with confocal laser scanning microscopy, merged fluorescence channels). First panel: Zera-EGFP co-expressed with BFP-fusions of the zeins listed on the left. Second panel: Zera-EGFP co-expressed with mCherry-fusions of the zeins listed on the left. Third panel: Zera-BFP co-expressed with EGFP-fusions of the zeins indicated on the left. Fourth panel: Zera-BFP co-expressed with mCherry-fusions of the zeins listed on the left. **(A-C)** Zera-XFP + 10-kDa-δ-zein-XFP, **(D-F)** Zera-XFP + 19-kDa-α-zein-XFP, **(G-J)** Zera-XFP + 22-kDa-α-zein-XFP, **(K-N)** Zera-XFP + 15-kDa-β-zein-XFP, **(O-R)** Zera-XFP + 16-kDa-γ-zein-XFP. Scale bar 5 µm. Individual/single channel data are presented in **Supplementary Figures S3**-**S6**.

To find out which of the zein-fusions would support the formation of PB-like structures on their own, we first expressed each of the zeins fused to EGFP individually (**Figure S2**). HDEL-tagged EGFP was used as control (**Figure S2A**). The EGFP fusions of 19-kDa-α-zein, 22-kDa-α-zein and 10-kDa-δ-zein, (**Figures S2B, C and F**) resulted mostly in diffuse signals and reticular structures with occasional small spots of more intense signal, resembling the picture observed with GFP-HDEL (**Figure S2A**). The EGFP fusions of 15-kDa-β-zein and 16-kDa-γ-zein resulted in patchy fluorescence patterns indicating small condensed structures with a diameter below 0.4 µm (**Figures S2D, E**). Only the expression of the EGFP-fusions with 27-kDa-γ-zein and Zera led to the formation of regular spherical particles with a diameter around or above 1 µm (**Figures S2G, H**). These results confirm that the N-terminal portion of the 27-kDa-γ-zein is vital and sufficient for the initiation of ectopic PBs in *N. benthamiana*, as expected. In order to obtain two-component PBs, we therefore continued with Zera, fused to either EGFP or BFP, and systematically tested it in combination with the other zeins, fused to mCherry or EGFP, respectively (**Figure 1**; **Supplementary Figures S3**, **S4**, **S5** and **S6**).

The 19-kDa- and 22-kDa-α-zein as well as 10-kDa-δ-zein fused to mCherry or EGFP accumulated separately from the Zera-induced PBs in all combinations of fluorescent labels examined (**Figures 1A-J**). However, the co-expression of fluorescently labelled 15-kDa-β-zein or 16-kDa-γ-zein together with Zera-XFP resulted in the formation of PBs containing both components (**Figures 1K-R**). Especially 15-kDa-β-zein-XFP was efficiently integrated into the Zera-induced PBs across all tested fluorophore combinations (**Figures 1K-N**). Switching fluorescent labels resulted in slightly different colocalization patterns, indicating some influence of the fusion partner on the exact spatial distribution of the components within the PBs (**Figures 1K-N**). Regardless of the variation in inclusions, only very little 15-kDa-β-zein-XFP remained outside the PBs in all cases, and the localization of 15-kDa-β-zein-XFP within PBs across different fluorophore combinations makes it a promising candidate for generating multi-layered PBs.

Like 15-kDa-β-zein, 16-kDa-γ-zein also localized within the matrix of Zera-induced PBs. However, this was not observed with all fluorescently tagged versions of 16-kDa-γ-zein (**Figures 1O-R**).

Based on these observations we concluded that 15-kDa-β-zein and 16-kDa-γ-zein interact most with the Zera-fusion protein. Consequently, they were selected for testing combinations of three components.

### 3.2 16-kDa-γ-zein promotes the generation of multi-layered protein bodies with three components in *N. benthamiana*

**Figure 2:**
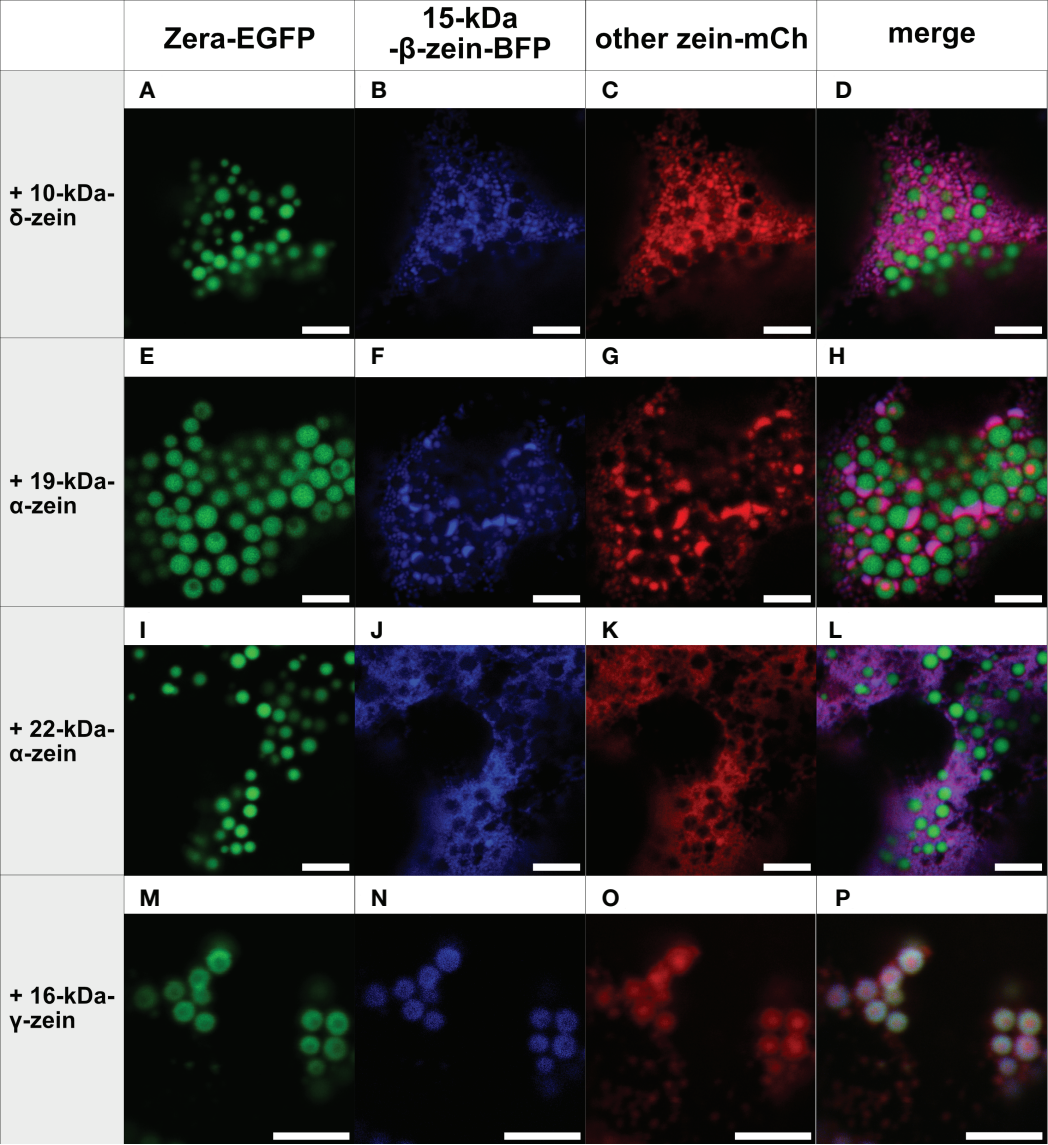
Co-expression of Zera-EGFP and 15-kDa-β-zein-BFP together with a third zein component fused to mCherry (representative images recorded with confocal laser scanning microscopy). First panel: Zera-EGFP; second panel: 15-kDa-β-zein-BFP; third panel: mCherry-fusion of the zeins listed on the left; fourth panel: overlay images. **(A-D)** Zera-EGFP + 15-kDa-β-zein -BFP +10-kDa-δ-zein-mCherry, **(E-H)** Zera-EGFP + 15-kDa-β-zein -BFP + 19-kDa-α-zein-mCherry, **(I-L)** Zera-EGFP + 15-kDa-β-zein -BFP + 22-kDa-α-zein -mCherry, **(M-P)** Zera-EGFP + 15-kDa-β-zein -BFP + 16-kDa-γ-zein-mCherry. Scale bar 5 µm.

In the next step, combinations of three zeins were co-expressed. **Figure 2** shows all combinations containing Zera-EGFP and 15-kDa-β-zein-BFP, together with one of the remaining zeins fused to mCherry. Interestingly, in the presence of 22-kDa-α-zein, 19-kDa-α-zein or 10-kDa-δ-zein, the 15-kDa-β-zein no longer integrated into Zera-induced PBs, but instead co-localized with these zein components outside of the ectopic PBs in an unstructured pattern, indicating an inhibition of the Zera/15-kDa-β-zein interactions by the other tested zeins, and possibly a stronger interaction with these zein components than with Zera (**Figures 2A-L**).

**Figure 3:**
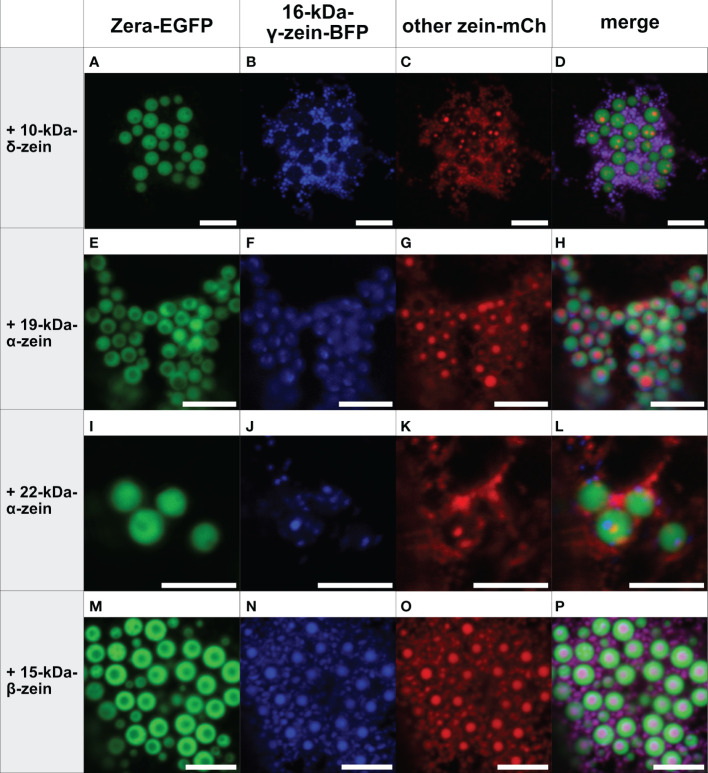
Co-expression of Zera-EGFP and 16-kDa-γ-zein-BFP together with a third zein component fused to mCherry (representative images recorded with confocal laser scanning microscopy). First panel: Zera-EGFP; second panel: 16-kDa-γ-zein-BFP; third panel: mCherry-fusion of the zeins listed on the left; fourth panel: overlay images. **(A-D)** Zera-EGFP + 16-kDa-γ-zein -BFP +10-kDa-δ-zein-mCherry, **(E-H)** Zera-EGFP + 16-kDa-γ-zein -BFP + 19-kDa-α-zein-mCherry, **(I-L)** Zera-EGFP + 16-kDa-γ-zein -BFP + 22-kDa-α-zein -mCherry, **(M-P)** Zera-EGFP + 16-kDa-γ-zein -BFP + 15-kDa-β-zein-mCherry. Scale bar 5 µm.

In contrast, the addition of 16-kDa-γ-zein-BFP to Zera-EGFP enabled the incorporation of 22-kDa-α-zein, 19-kDa-α-zein or 10-kDa-δ-zein into ectopic PBs (**Figure 3**), despite these components not colocalizing with Zera-induced PBs by themselves (**Figures 1A-J**). While the incorporation was more efficient in case of 19-kDa-α-zein (**Figure 3H**), it was only partial, but clear in case of 22-kDa-α-zein and 10-kDa-δ-zein (**Figures 3D, L**). As expected, the combination of Zera-EGFP and 16-kDa-γ-zein-BFP with 15-kDa-β-zein-mCherry, which would already integrate into Zera-induced PBs on its own (**Figure 1L**), also led to efficient inclusion of the 15-kDa-β-zein-fusion (**Figure 3P**), confirming the result shown above with a different combination of fluorophores (**Figures 2M-P**).

As a control, we co-expressed Zera-GFP and 16-kDa-γ-zein-BFP together with secretory mCherry (lacking the 15-kDa-β-zein moiety) to exclude the possibility, that incorporation into protein bodies was due to unspecific interactions of the fluorescent proteins. Secretory mCherry was detected in the apoplast (**Figure S7**), and was not incorporated into protein bodies, indicating that an integration into multi-layered PBs was indeed due to the respective zein portions and specific interactions between them.

Overall, these results indicate a crucial role of 16-kDa-γ-zein in incorporating other zeins into Zera-induced ectopic PBs, thus promoting the formation of multi-layered PBs containing three different components. With some combinations, this mediating role of 16-kDa-γ-zein was further highlighted by its presence at the interface between the shell formed by Zera-EGFP, and the inclusions in the core (**Figure S8**).

While the combination of Zera and 16-kDa-γ-zein with both 19-kDa-α-zein and 15-kDa-β-zein resulted in PBs with a core-shell structure, the combination of Zera, 16-kDa-γ-zein, and 15-kDa-β-zein led to particularly efficient and reliable generation of multi-layered PBs. Therefore, this combination was selected for further experiments.

### 3.3 Multi-layered protein bodies remain intact upon isolation

Next, we addressed the question if the interaction between the zein components is strong enough such that the multi-layered structure of the particles stays intact upon isolation of the PBs from the leaf material. For this, we applied an isolation procedure based on homogenization and sonication followed by a series of filtrations with decreasing cut-offs and a centrifugation step. Two-component and three-component PBs consisting of Zera-EGFP and 15-kDa-β-zein-mCherry or Zera-EGFP, 16-kDa-γ-zein-BFP and 15-kDa-β-zein-mCherry, respectively, were then analyzed by confocal microscopy. In both cases, the multi-component PBs were still intact, suggesting strong and durable zein-zein interactions within the PBs (**Figure S8** and **S9**).

**Figure 4:**
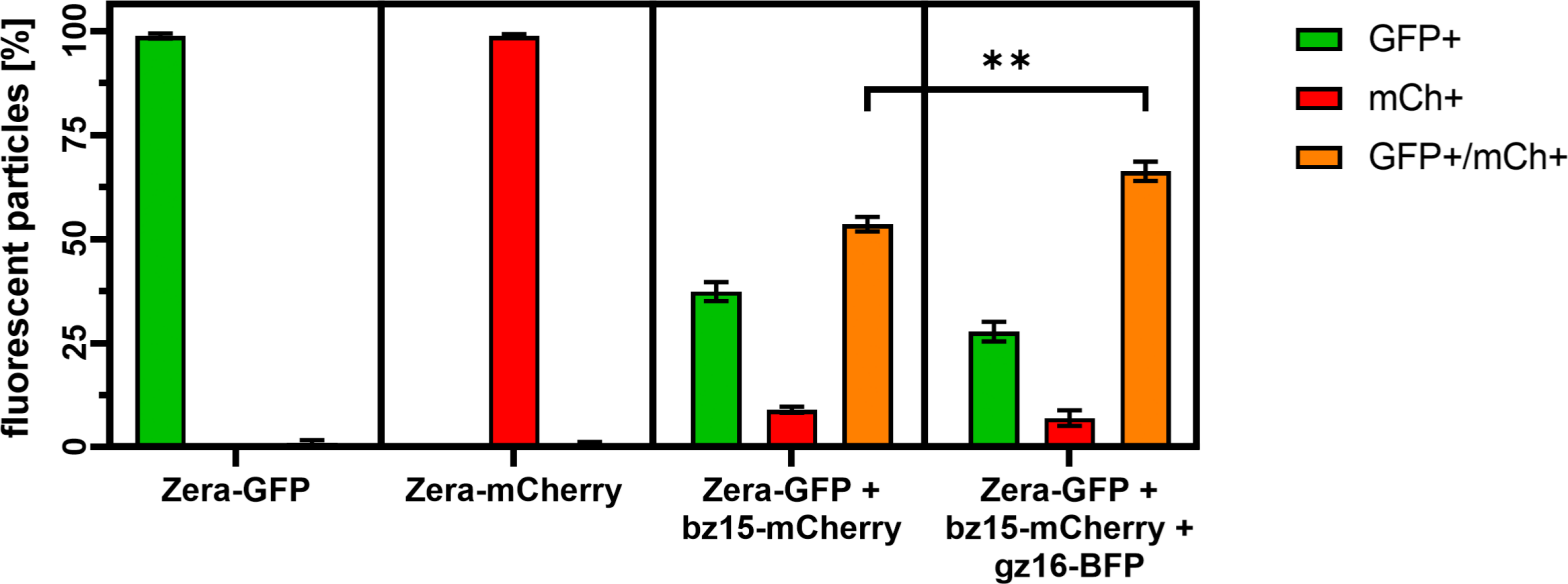
Flow cytometry measurement of isolated protein bodies resulting from the expression of a single zein-fusion (Zera-GFP or Zera-mCherry), the co-expression of Zera-GFP and 15-kDa-β–zein-mCherry (Zera-GFP + bz15-mCherry), or the co-expression of Zera-GFP, 15-kDa-β–zein-mCherry and 16-kDa-γ-zein-BFP (Zera-GFP + bz15-mCherry + gz16-BFP). The percentage of GFP-positive (GFP+, green bar), mCherry-positive (mCh+, red bar), and double-positive (GFP+/mCh+, orange bar) particles is shown. Values for three independent measurements are shown. A t-test was performed, p > 0.05, ** p ≤.01.

To confirm this result and to quantify the rate of incorporation of 15-kDa-β-zein-mCherry into the PB-core, the particle preparations were analyzed by flow cytometry (**Figure 4**). As expected, PBs containing only Zera-EGFP were detectable as single green fluorescent particles, whereas a high proportion of isolated PBs resulting from co-expression of Zera-EGFP and 15-kDa-β-zein-mCherry were detectable as double fluorescent particles (**Figure 4**), indicating the inclusion of 15-kDa-β-zein-mCherry into Zera-EGFP PBs. When 16-kDa-γ-zein-BFP was co-expressed in addition, we could determine a further 10% increase in PBs incorporating 15-kDa-β-zein-mCherry (**Figure 4**), confirming the role of 16-kDa-γ-zein in promoting the formation of multi-layered PBs.

### 3.4 Encapsulation of a pharmaceutically relevant protein into multi-layered protein bodies

**Figure 5:**
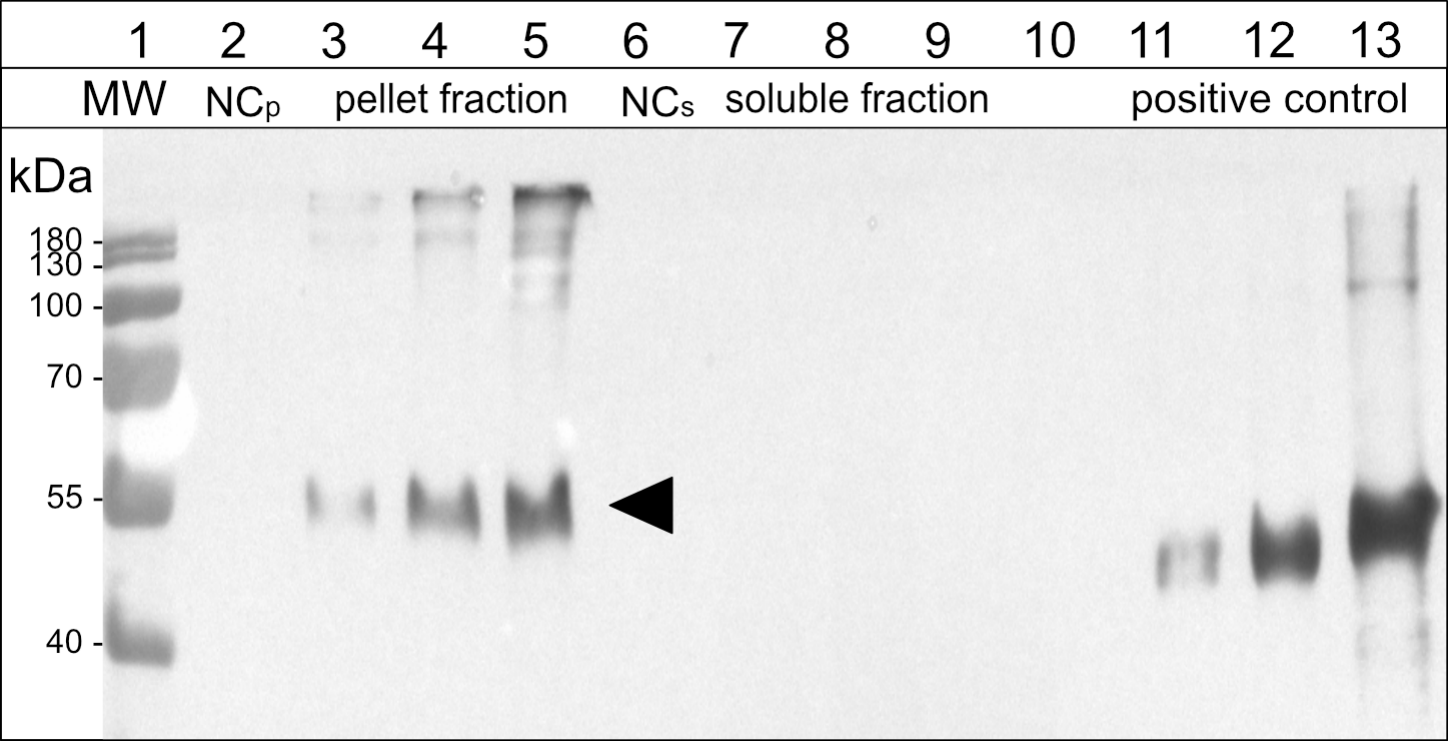
Immunoblot analysis of the soluble and pellet fractions obtained from agroinfiltrated leaf samples expressing the antigen-fusion (15-kDa-β-zein-Opt3a12-flag). Lane 2-5: pellet fraction, solubilized with reducing and denaturing buffer; Lane 6-9: soluble fraction, extracted in saline buffer containing non-ionic detergent. Lanes 3 and 7: 15-kDa-β-zein-antigen fusion expressed on its own. Lanes 4 and 8: 15-kDa-β-zein-antigen fusion when co-expressed with Zera-EGFP. Lanes 5 and 9: 15-kDa-β-zein-antigen fusion when co-expressed with Zera-EGFP and 16-kDa-γ-zein-BFP. NCp and NCs – pellet or soluble fraction, respectively, obtained from leaves infiltrated with an empty vector control. Lane 11-13, positive control (200, 500 and 1500 ng of a flag-tagged standard protein). MW – protein ladder. Bands corresponding to the 15-kDa-β-zein-Opt3a12-flag fusion with a molecular mass of approximately 50 kDa are indicated by an arrow. The estimates of relative quantities were based on three blots using extracts from three independent infiltration experiments.

To confirm our results with a pharmaceutically relevant model antigen for a potential vaccine, we used a peptide, derived from the outer membrane protein BB0172 of *B. burgdorferi*, the pathogen that is the causative agent of Lyme disease (Wood et al., 2013; Small et al., 2014). The N-terminal addition of 15-kDa-β-zein to the antigen (**Figure S1C**) resulted in the expression of sufficiently large amounts of fusion protein that were readily detectable by immunoblot analysis (**Figure 5**, lane 3), whereas fusing the antigen to Zera did not lead to the formation of ectopic PBs, and the fusion protein did not accumulate to detectable amounts.

**Figure 6:**
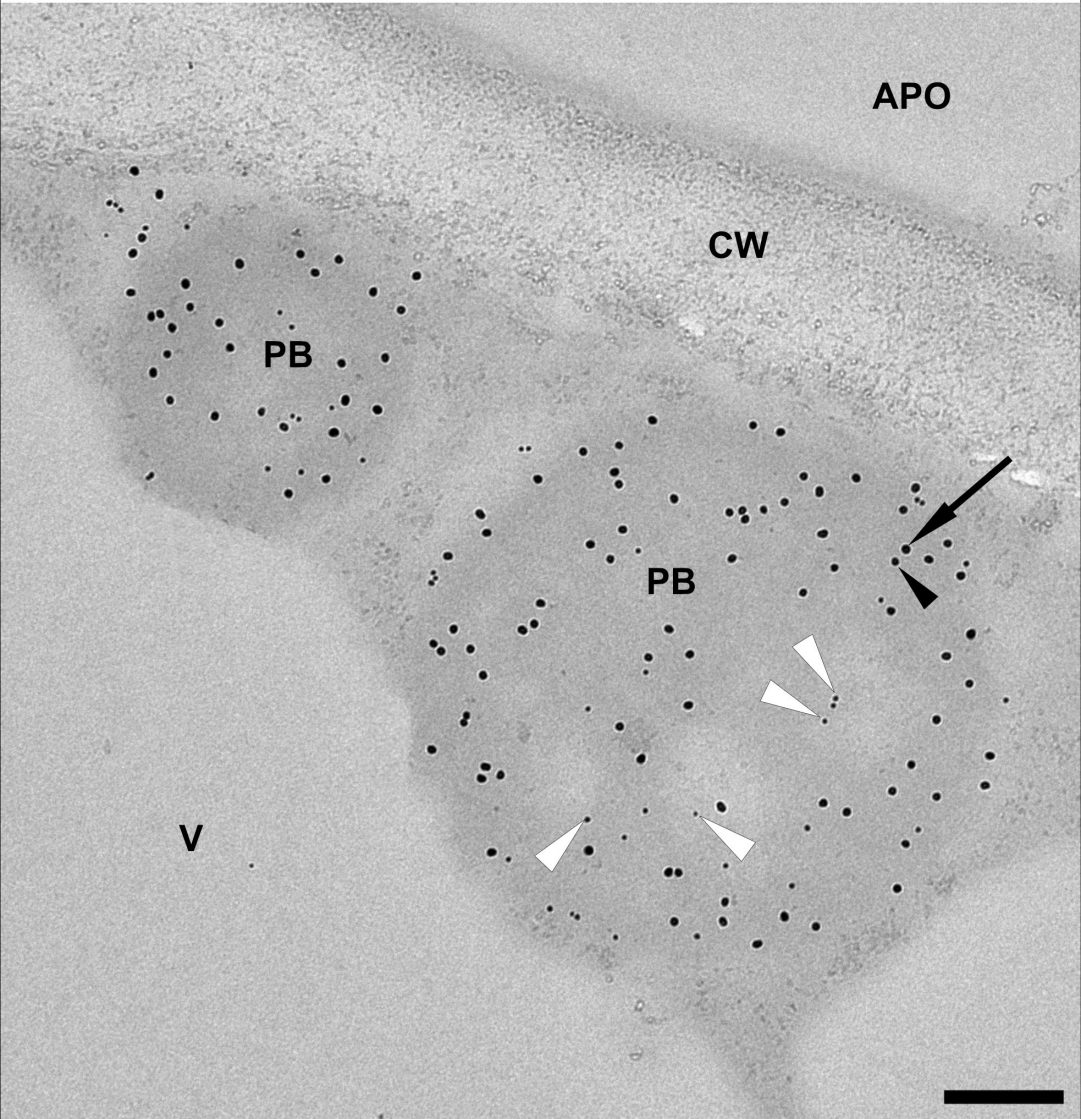
Representative electron microscopy image indicating the incorporation of the model antigen into multi-component PBs. Immunogold labelling of an infiltrated leaf sample shows protein bodies (PB) composed of Zera-EGFP (18 nm gold particles, arrow), 16-kDa-γ-zein-BFP (10 nm gold particles, black arrowhead) and 15-kDa-β-zein- Opt3a12-flag (6 nm gold particles, white arrowheads). Note that the 6 nm particles, corresponding to the localization of the antigen, are preferentially found in the lighter areas of the PB. Protein body (pb), apoplast (apo), cell wall (cw), vacuole (v). Scale bar 0.25 µm.

To incorporate the antigen fusion into the core of the induced PBs, we co-expressed Zera-EGFP and 16-kDa-γ-zein-BFP alongside 15-kDa-β-zein-antigen. Since the 15-kDa-β-zein-antigen construct did not possess fluorescent properties, immunogold labelling and subsequent transmission electron microscopy of embedded agroinfiltrated leaf sections was used to detect all three zein-fusion proteins in a single PB (**Figure 6**). The largest gold particles (18 nm), indicating localization of Zera-EGFP, are more abundant in the electron-dense regions at the periphery of the PB, whereas the smallest gold particles (6 nm), indicating the localization of the Flag-tagged antigen fused to 15-kDa-β-zein, are mostly found in the electron-lucent regions towards the center of the PB (**Figure 6**, white arrowheads). The medium size gold particles (10 nm) indicate the position of the 16-kDa-γ-zein-BFP. Overall, these immunolocalization results confirm the encapsulation of the antigen-fusion within the PBs.

The effect of zein co-infiltrations on the accumulation of the 15-kDa-β-zein-antigen fusion protein was investigated by protein extraction and subsequent immunoblotting. Infiltrated leaves were extracted in saline buffer containing non-ionic detergent, pelleted, and then pellets were re-extracted with a strongly reducing buffer supplemented with 1 M urea and 7 M thiourea. This enabled the separation and individual analysis of the insoluble and soluble fraction of the extract. When the 15-kDa-β-zein-antigen fusion was expressed by itself (without the co-expression of additional zeins), all the antigen was recovered in the pellet fraction indicating the formation of insoluble polymers. Furthermore, antigen cleavage from the zein moiety was not observed (**Figures 5**, lanes 3 and 7). The additional co-expression of Zera-EGFP causing the incorporation of the 15-kDa-β-zein-antigen fusion into Zera-induced PBs, resulted in a marked increase in antigen accumulation (**Figure 5**, lane 4), highlighting the positive effect of encapsulation on antigen yield. Additional co-expression of 16-kDa-γ-zein further increased the antigen content to approximately 259 ( ± 91) mg/kg leaf FW (**Figure 5**, lane 5). This supports the data gathered by flow cytometry and confirms the beneficial role of 16-kDa-γ-zein in the integration of 15-kDa-β-zein into Zera-induced PBs, hence promoting the formation of multi-layered PBs.

## 4 Discussion

Overall, our study demonstrates that it is possible to produce multicomponent ectopic PBs by transient co-expression of selected zeins in *Nicotiana benthamiana*, highlighting that the interactions between the storage protein components are both necessary and sufficient to achieve an orderly composition resembling the model of naturally occurring zein PBs in maize. This in turn emphasizes that native PBs are supramolecular structures formed by a complex interplay of storage proteins that self-assemble into spherical particles in the lumen of the ER through reversible interactions (Lending and Larkins, 1989; Pedrazzini et al., 2016). Unlike aggregation, which describes a process of irreversible binding of misfolded proteins, this process rather resembles coalescence or similar phenomena, which sometimes serve to protect parts of the proteome from degradation (Saarikangas and Barral, 2016). The prerequisite for the synthetic production of multi-layered PBs for bioencapsulation is therefore the understanding of the interaction of individual zeins as their building blocks (Guo et al., 2013). These building blocks comprise α-, β-, γ-, and δ-types of zein, of which the β-, γ-, and δ-types are usually encoded by single-copy genes and include one 15-kDa β-type zein (Pedersen et al., 1986), two δ-type zeins of 10- and 18-kDa (Kirihara et al., 1988; Chui and Falco, 1995), and three γ-type zeins of 16-, 27-, and 50-kDa (Prat et al., 1987; Woo et al., 2001; Li and Song, 2020). The α-zein subfamily can be further divided into 19- and 22-kDa subclasses which are encoded by multiple gene families (Wilson and Larkins, 1984). The functions of individual zeins in PB biogenesis have been studied in their native environment, using either natural mutants or knockdown lines (Segal et al., 2003; Wu and Messing, 2010; Guo et al., 2013). Although these studies provided important information on the localization and behavior of zeins, they could not analyze the role of individual zeins independently of other endogenous factors. A yeast two-hybrid study provided further important insights into direct zein interactions (Kim et al., 2002), but could not account for the effects of their spatial arrangement in the PB matrix. Other studies investigated if zeins accumulate into combined particles upon ectopic expression, but did not determine the localization of individual zeins (Bagga et al., 1997; Coleman et al., 2004; Kim and Krishnan, 2019). The additive approach in the present study aimed to reveal the individual positioning of different zeins upon ectopic co-expression. While this provides valuable insight into the mechanisms of storage organelle formation in seeds, it is important to note that the environment is not necessarily representative of the native state in all respects. For example, native PBs contain numerous non-zein proteins (NZPs) in addition to zeins, including FLOURY1 (FL1) and OPAQUE10 (O10) (Holding et al., 2007; Wang et al., 2016). Moreover, in maize seeds, other mechanisms in addition to protein-protein interactions between zeins have been identified as an important factor for the structural organization of PBs. For example, altered mRNA targeting can disrupt the localization pattern of zeins in maize PBs (Washida et al., 2004; Washida et al., 2009). The intricate temporal regulation of zein expression and the resulting relative abundance and stoichiometric ratio of zeins also contribute to the mechanism of endogenous PB formation (Guo et al., 2013; Li and Song, 2020). Nevertheless, the study of interactions between PB resident proteins in a heterologous system is a valuable complementary approach, and this was recently pursued to show that the accumulation of NZP1 in induced PB depends on its interaction with 22-kDa-α-zein (Feng et al., 2022).

Our data confirm that among the zeins, the N-terminal part of 27-kDa-γ-zein (commercially developed as Zera) is necessary a for the initiation of PBs when fused to other polypeptides (Geli et al., 1994; Torrent et al., 2009a; Wu and Messing, 2010). The 27-kDa-γ-zein is the evolutionary oldest zein, and while its C-terminal part seems to be derived from 2-S-albumin, the N-terminal part with amphipathic repeats of the amino acid sequence PPPVHL promotes both membrane interaction and spontaneous self-assembly (Kogan et al., 2001) which are crucial for ER-retention. Additionally, seven cysteine residues drive polymerization and stabilize the resulting supramolecular structure *via* disulfide bridges (Geli et al., 1994; Torrent et al., 2009b; Mainieri et al., 2014). It has been previously shown that the formation of ectopic PBs can be triggered by Zera (Mainieri et al., 2004; Torrent et al., 2009b), but the potential for organization into stratified PBs in a heterologous expression system has not been studied in detail.

During native PB biogenesis in maize endosperm, 27-kDa-γ-zein interacts strongly with 15-kDa-β-zein and 16-kDa-γ-zein, which are present in the periphery of newly formed PBs and locate closer to the center at later stages of native PB biogenesis (Lending and Larkins, 1989). In the heterologous system, both 15-kDa-β-zein and 16-kDa-γ-zein co-localized very well with Zera-induced PBs, ranging from patches at the periphery of the PB, to a homogeneous distribution or even a separation into a core-shell structure within the PB. This diversity of co-localization behavior reflects to some extent their differential distribution during native PB maturation. Without co-expression of Zera the EGFP fusions of 15-kDa-β-zein or 16-kDa-γ-zein did not form regular, spherical PBs, but resulted in patchy fluorescence patterns indicating small protein aggregates. This is well in agreement with earlier observations in Arabidopsis leaves where 16-kDa-γ-zein formed dispersed electron-dense threads enlarging the ER lumen without assembling into PBs, and only when co-expressed with 27-kDa-γ-zein it co-assembles with the latter into insoluble polymers (Mainieri et al., 2018). In transgenic Arabidopsis seeds 16-kDa-γ-zein formed more compact aggregates, but remained largely insoluble in reducing conditions, also suggesting that 16-kDa-γ-zein is unable to form well-ordered polymers on its own but relies on the ability of 27-kDa-γ-zein to drive self-assembly (Arcalis et al., 2021). Although 16-kDa-γ-zein most likely evolved through gene duplication of 27-kDa-γ-zein (Xu and Messing, 2009), this different behavior may be explained by the loss of the repeat region as well as some cysteine residues reducing the strength of its intermolecular interactions (Mainieri et al., 2018). A similar explanation may apply to 15-kDa-β-zein, which lacks the amphipathic repeats, while still showing a sequence similarity of 85% to the N-terminal region of other proteins of the γ-zein family (Kim et al., 2002). However, when co-expressed with Zera-EGFP, 15-kDa-β-zein-mCherry efficiently contributed to two-component PBs, and the combination of these two zeins resulted in the desired core-shell structure of the PBs.

In contrast, the fluorescently labeled 10-kDa-δ-zein exhibited only rare and small inclusions in PBs, when co-expressed with Zera. In native PBs 10-kDa-δ-zein is found together with 19-kDa-α-zein in the PB core, but neither appears to directly interact with γ-zeins in the shell (Kim et al., 2002). The observation of occasional co-localization is consistent with a TEM analysis that indicated the formation of mixed PBs upon co-expression of δ-zeins with 27-kDa-γ-zein in soybean, as inferred from electron density (Kim and Krishnan, 2019).

We observed that the co-expressed α-zeins (19-kDa-α-zein and 22-kDa-α-zein) that are located in the core and interface of native PBs were clearly localized separately from the Zera-induced PBs when expressed individually, with 19-kDa-α-zein being incorporated only in very rare cases. This is consistent with data from a yeast two-hybrid study showing no direct interaction with 27-kDa-γ-zein (Kim et al., 2002). Consequently, it was speculated that other zeins and non-zein proteins may be required for their incorporation, and it has been shown, for example, that 22-kDa-α-zein remains outside of PBs in the absence of the membrane protein floury1 (Holding et al., 2007).

Among the zeins, 16-kDa-γ-zein and 15-kDa-β-zein have been suggested to play a mediating role between other PB components because they interact with most other zeins (Kim et al., 2002). Suppression studies also indicated that 16-kDa-γ-zein or 15-kDa-β-zein might play a role in integrating other zeins (Wu and Messing, 2010; Guo et al., 2013). Indeed, a strong effect on the integration of other zeins into PBs was observed when 16-kDa-γ-zein was co-expressed, making it a key element in the design of synthetic PBs. Together with 15-kDa-β-zein, 16-kDa-γ-zein formed combined homogeneous inclusions within Zera PBs that were larger and more abundant than those of 15-kDa-β-zein alone, which was also confirmed by flow cytometry analysis of isolated PBs. Co-expression of 16-kDa-γ-zein even allowed integration of 19-kDa-α-zein, 22-kDa-α-zein, and 10-kDa-δ-zein into Zera PBs, albeit to a lesser extent. Moreover, 16-kDa-γ-zein was also observed at the interface between the PB core and the shell formed by Zera, consistent with its role as a linker.

It should be noted that co-infiltration of individual agrobacteria lines each carrying a single zein construct was used in this study to facilitate the screening of a large number of zein combinations. This approach however is prone to causing some variation in expression levels within the leaf (Bashandy et al., 2015). For future large-scale production the construction of a single vector, which contains all the required genes or their stable integration into the plant genome would therefore be favorable to increase the uniformity of PBs within a single cell and throughout the leaf tissue. For example, according to our results a platform could be envisaged, that stably expresses both Zera and 16-kDa-γ-zein at an optimized stoichiometric ratio, providing the chassis for bioencapsulation. A therapeutic protein could then be incorporated by fusing it to 15-kDa-β-zein or one of the other zein candidates identified and adding it to the expression host *via* agroinfiltration. This would allow the on-demand production of, for example, seasonal or emerging vaccine antigens.

The fact, that the multicomponent PBs can be isolated and retain their internal architecture further suggests, that various proteins of interest can be stably incorporated and accumulated by this approach. To confirm that multicomponent PBs can also be achieved with a pharmaceutically relevant model antigen, we used a peptide derived from *B. burgdorferi* outer surface protein BB0172, which was previously identified as a promising veterinary vaccine candidate for protection against Lyme disease (Small et al., 2014). This antigen did not accumulate to detectable levels when conventionally fused to Zera, but in fusion with 15-kDa-β-zein it was clearly detectable. As expected, fusion with 15-kDa-β-zein rendered the antigen insoluble, and no free or soluble antigen was present. After co-expression of Zera-EGFP, which resulted in incorporation of the 15-kDa-β-zein-fused antigen into Zera-induced PBs, the antigen accumulated to higher levels, revealing a positive effect of encapsulation on antigen yield. Additional co-expression of 16-kDa-γ-zein further doubled the antigen content. This confirms a positive role of 16-kDa-γ-zein in protecting the 15-kDa-β-zein-fusion protein within Zera-induced PBs. Our immunolocalization results confirmed the incorporation of antigen fused with 15-kDa-β-zein into multicomponent PBs. Previous studies have shown that the BB0172 derived peptide antigen is immunogenic but requires potent adjuvants to be effective (Small et al., 2014). Since the delivery of subunit antigens on particulate structures is an established approach to enhance their immunogenicity (Rybicki, 2020; Ximba et al., 2022), we anticipate that through encapsulation in PBs, the vaccine candidate will benefit not only from higher yield but also from the adjuvant effect of PBs. Further studies will be needed to confirm this.

In summary, the *in planta* encapsulation of antigens within newly designed multi-layered protein storage organelles with a core-shell structure is a novel approach that can be integrated in the upstream production process to enhance the accumulation and to achieve a particulate formulation. In future, this strategy may allow to further maximise the benefits of particulate plant-based drug delivery vehicles by functionalizing the individual PB components in different ways, for example combining the antigen, a receptor-binding ligand and an adjuvant in one particle.

## Data availability statement

The raw data supporting the conclusions of this article will be made available by the authors, without undue reservation.

## Author contributionss

JS, MT, LZ and ES designed and carried out experiments, analyzed data, and wrote the manuscript. EA and FS designed and carried out experiments and analyzed data. ME-G, EP and AV discussed the data, and contributed to the manuscript. JS, LZ and ES designed the study, analyzed data, and wrote the manuscript. All authors contributed to the article and approved the submitted version.
