# Supplementary material for: Generation of multi-layered protein bodies in N. benthamiana for the encapsulation of vaccine antigens
Source: Front Plant Sci. 2023 Jan 17;14:1109270. doi: 10.3389/fpls.2023.1109270 (PMC9887037; doi:10.3389/fpls.2023.1109270)
Supplement: Supplementary file 1 [file DataSheet_1.pdf]

Supplementary Material

Generation of multi-layered protein bodies in *N. benthamiana* for the encapsulation of vaccine antigens

Jennifer Schwestka[1*], Lukas Zeh[1], Marc Tschofen[1], Fabian Schubert[1], Elsa Arcalis[1], Maria Esteve-Gasent[2], Emanuela Pedrazzini[3], Alessandro Vitale[3], Eva Stoger[1]

*** Correspondence:** Corresponding Author: Jennifer.schwestka@boku.ac.at

1 Supplementary Figures

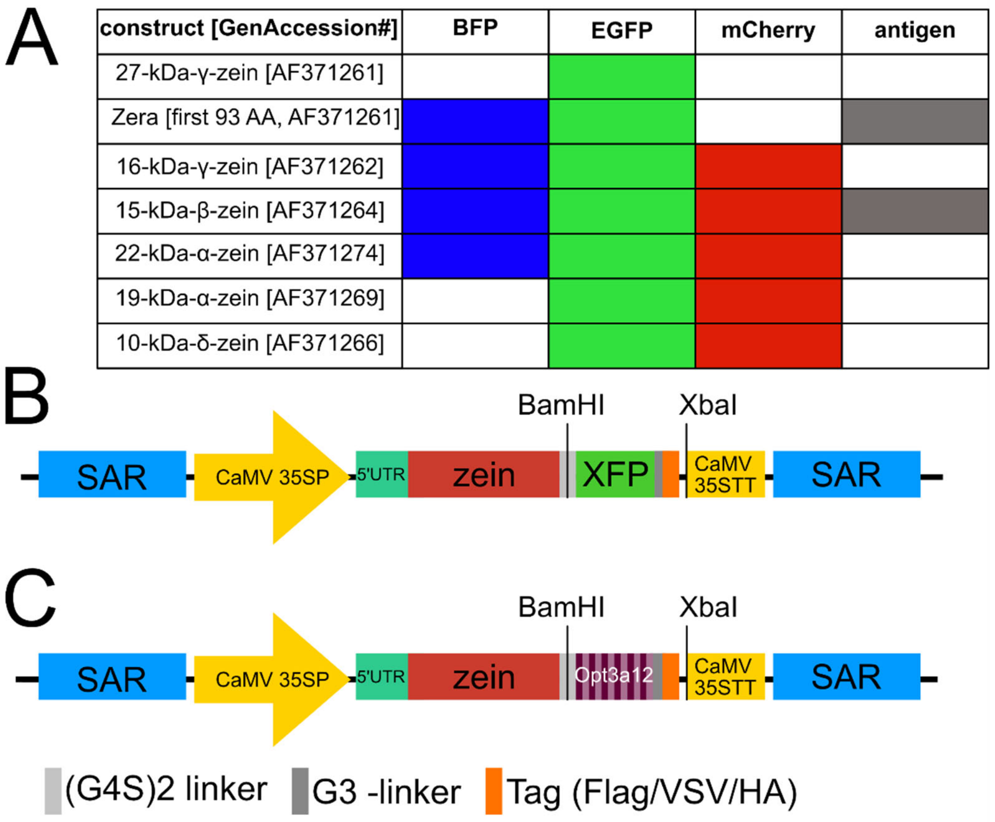

Supplementary Figure S1. Plant expression cassette and construct design. Schematic representation of the different constructs and the expression cassette of the plant binary expression vector pTRA. (A) Overview of the different zein-fusion constructs available in this study. (B) Cassette containing the different zein-XFP constructs. (C) Construct containing the zein-antigen (Opt3a12) fusion. Opt3a12 consists of 12 repeats of a derivative of peptide B originating from the Borrelial outer membrane protein BB0172 (Hassan et al., 2019). The restriction sites used to insert the fluorescent tag or the gene of interest into the plant expression vector are indicated. SAR: Scaffold attachment

region; CaMV 35SSP/TT: promoter with duplicated enhancer and terminator of the Cauliflower mosaic virus (CaMV) 35S gene; 5'UTR: untranslated region of the tobacco etch virus; zein: Zera, 16-kDa-γ-, 15-kDa-β-, 10-kDa-δ-, 19-kDa-α- or 22-kDa-α-zein; XFP: EGFP, mCherry, or mTagBFP2; grey box: glycine-rich linker; orange box: tags for detection (Flag-, VSV- or HA-tag).

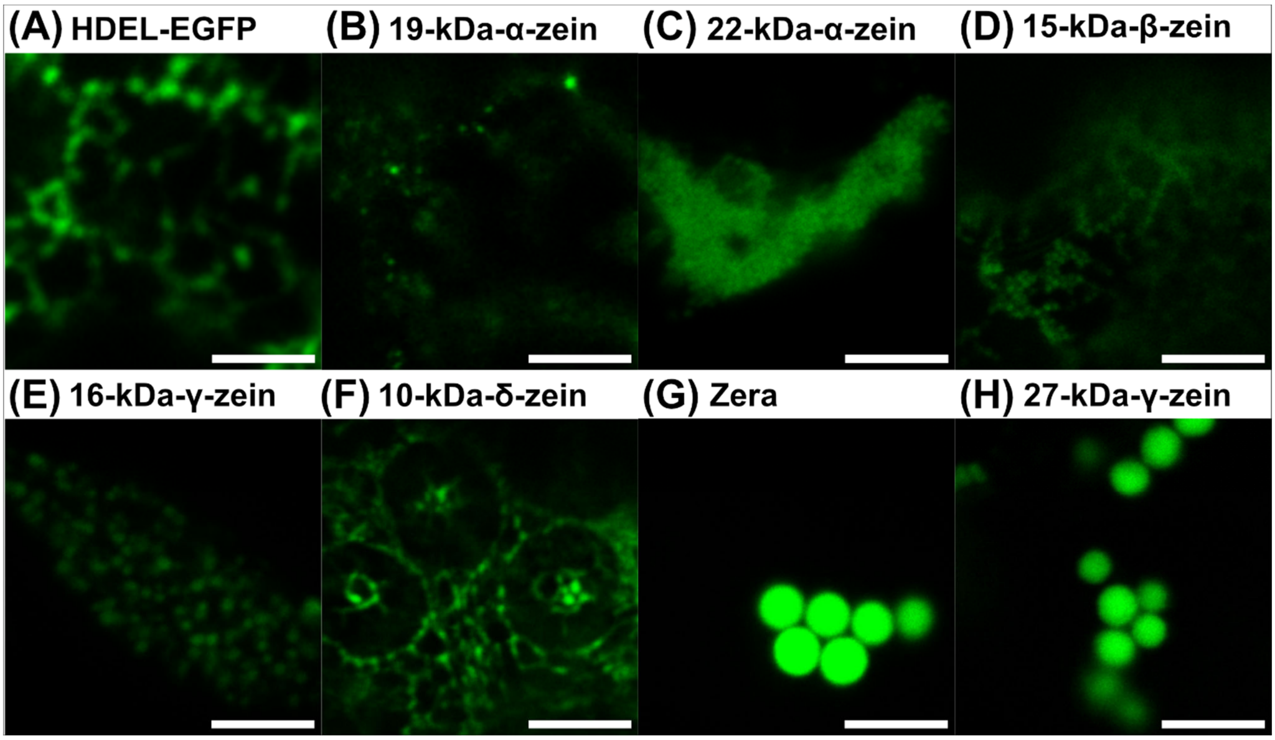

Supplementary Figure S2. Overview of the fluorescent signal distribution after transient expression of the different zein-EGFP fusions in *N. benthamiana*. (A) HDEL-EGFP, (B) 19-kDa-α-zein-EGFP, (C) 22-kDa-α-zein-EGFP, (D) 15-kDa-β-zein-EGFP, (E) 16-kDa-γ-zein-EGFP, (F) 10-kDa- δ-zein-EGFP, (G) Zera-EGFP and (H) 27-kDa-γ-zein-EGFP. Scale bar 5 µm.

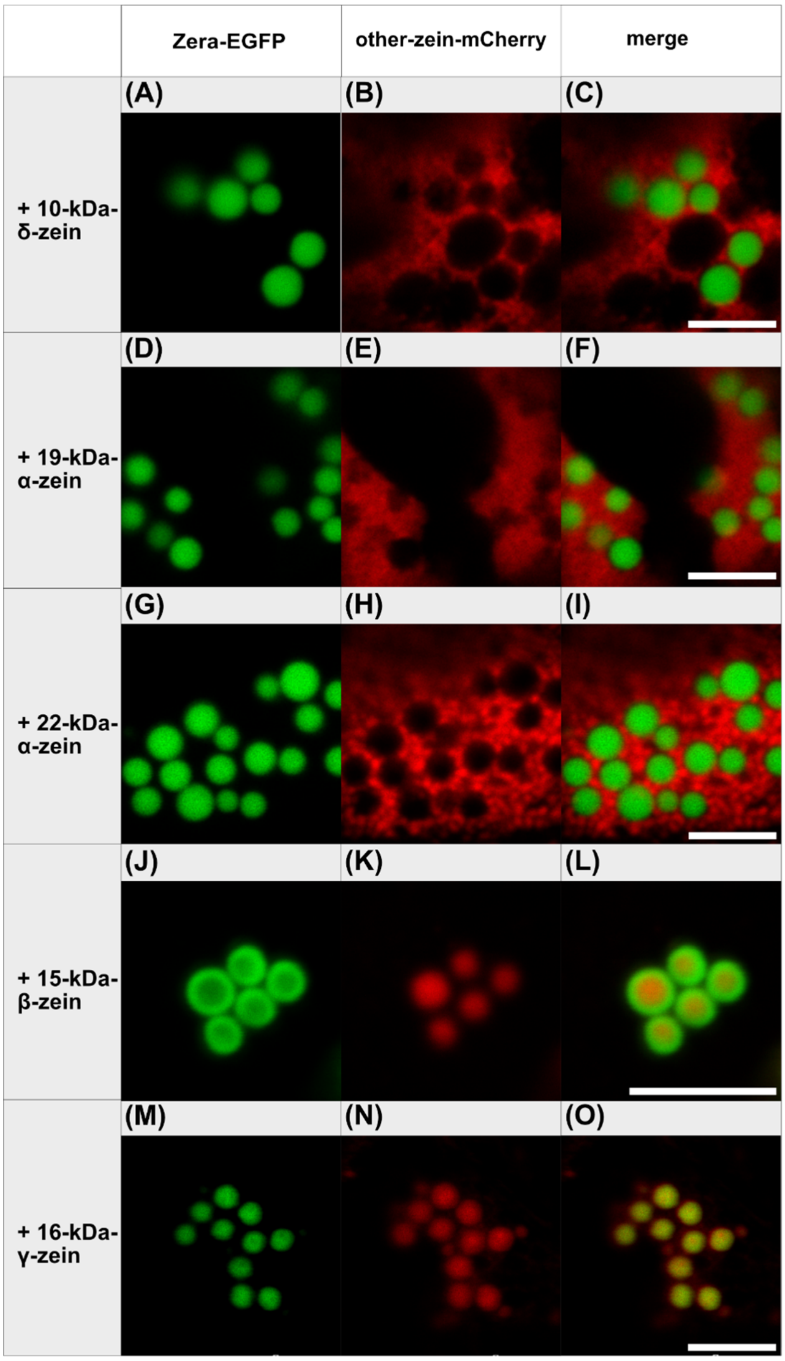

Supplementary Figure S3. Co-expression of Zera-EGFP with zein-mCherry fusion constructs (individual/single channel data for merged images shown in Figure 1). First panel: Zera-EGFP. Second panel: zein-mCherry-fusion of the zeins listed on the left. Third panel: overlay images. (A-C) Zera-EGFP + 10-kDa-δ-zein-mCherry, (D-F) Zera-EGFP + 19-kDa-α-zein-mCherry, (G-I) Zera-EGFP + 22-kDa-α-zein-mCherry, (J-L) Zera-EGFP + 15-kDa-β-zein-mCherry, (M-O) Zera-EGFP + 16-kDa-γ-zein-mCherry. Scale bar 5 µm.

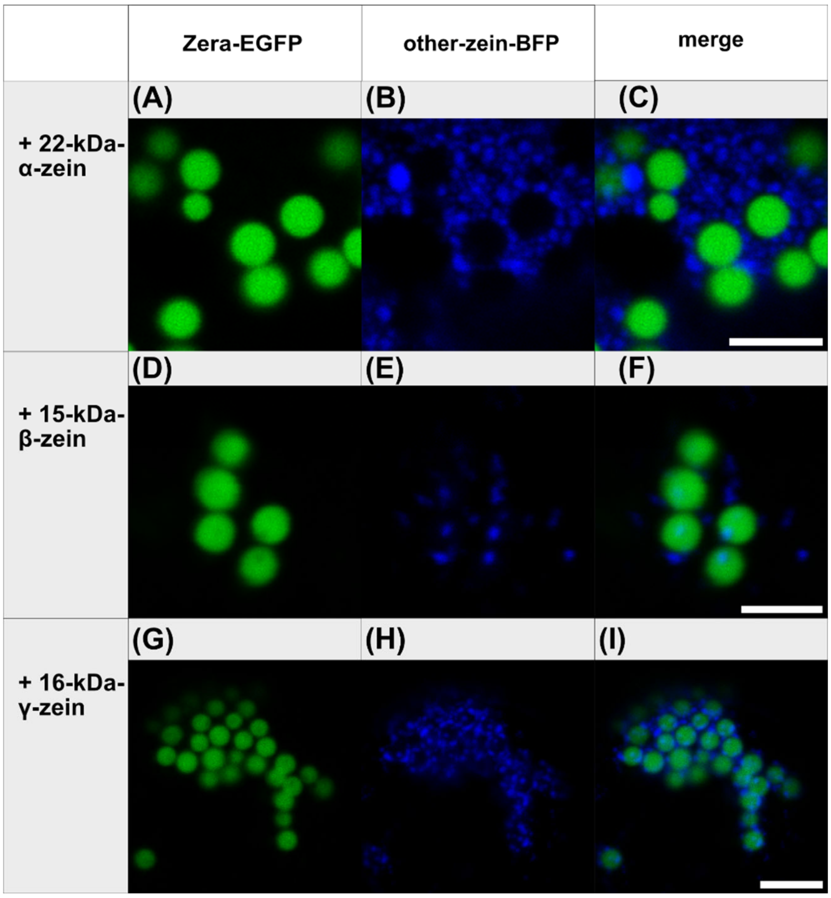

Supplementary Figure S4. Co-expression of Zera-EGFP with zein-BFP constructs (individual/single channel data for merged images shown in Figure 1). First panel: Zera-EGFP; second panel: zein-BFP-fusion of the zeins listed on the left. Third panel: overlay images. (A-C) Zera-EGFP + 22-kDa-α-zein-BFP, (D-F) Zera-EGFP + 15-kDa-β-zein-BFP, (G-I) Zera-EGFP + 16-kDa-γ-zein-BFP. Scale bar 5 μm.

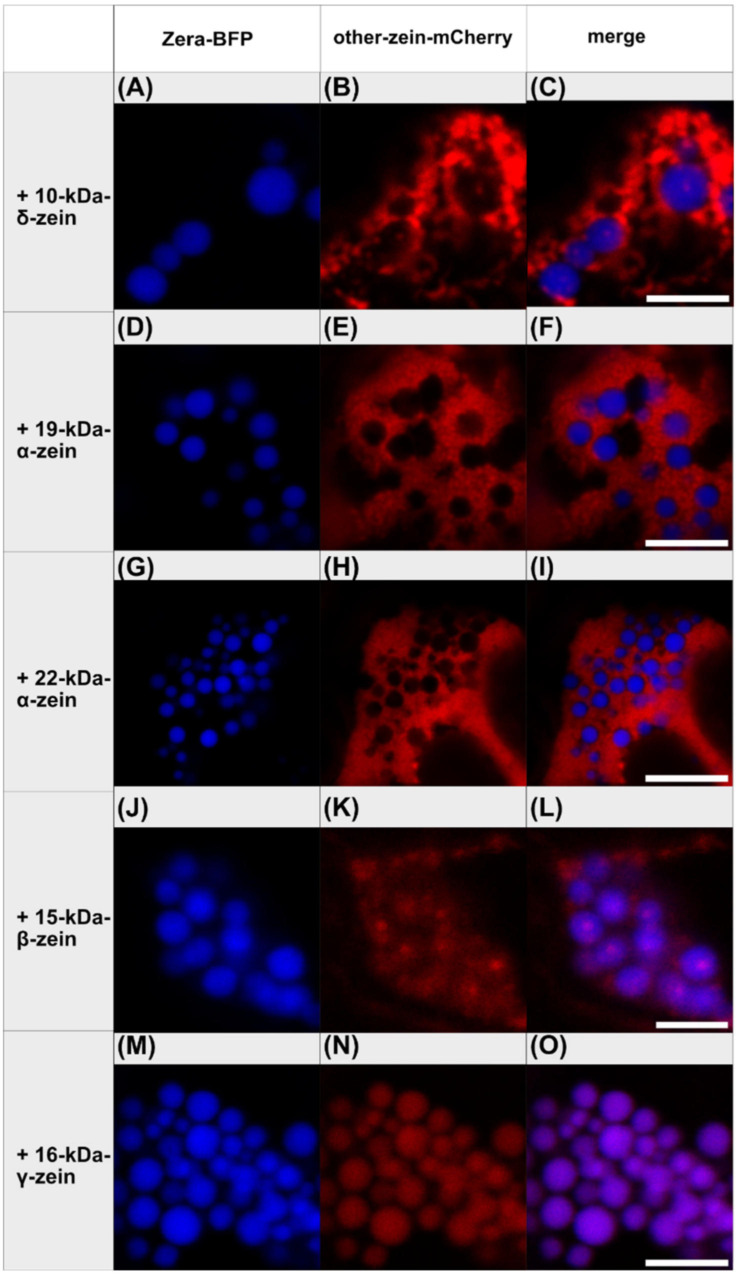

Supplementary Figure S5. Co-expression Zera-BFP with zein-mCherry constructs (individual/single channel data for merged images shown in Figure 1). First panel: Zera-BFP; second panel: zein-mCherry-fusion of the zeins listed on the left. Third panel: overlay images. (A-C) Zera-BFP + 10-kDa-δ-zein-mCherry, (D-F) Zera-BFP + 19-kDa-α-zein-mCherry, (G-I) Zera-BFP + 22-kDa-α-zein-mCherry, (J-L) Zera-BFP + 15-kDa-β-zein-mCherry, (M-O) Zera-BFP + 16-kDa-γ-zein-mCherry. Scale bar 5 µm.

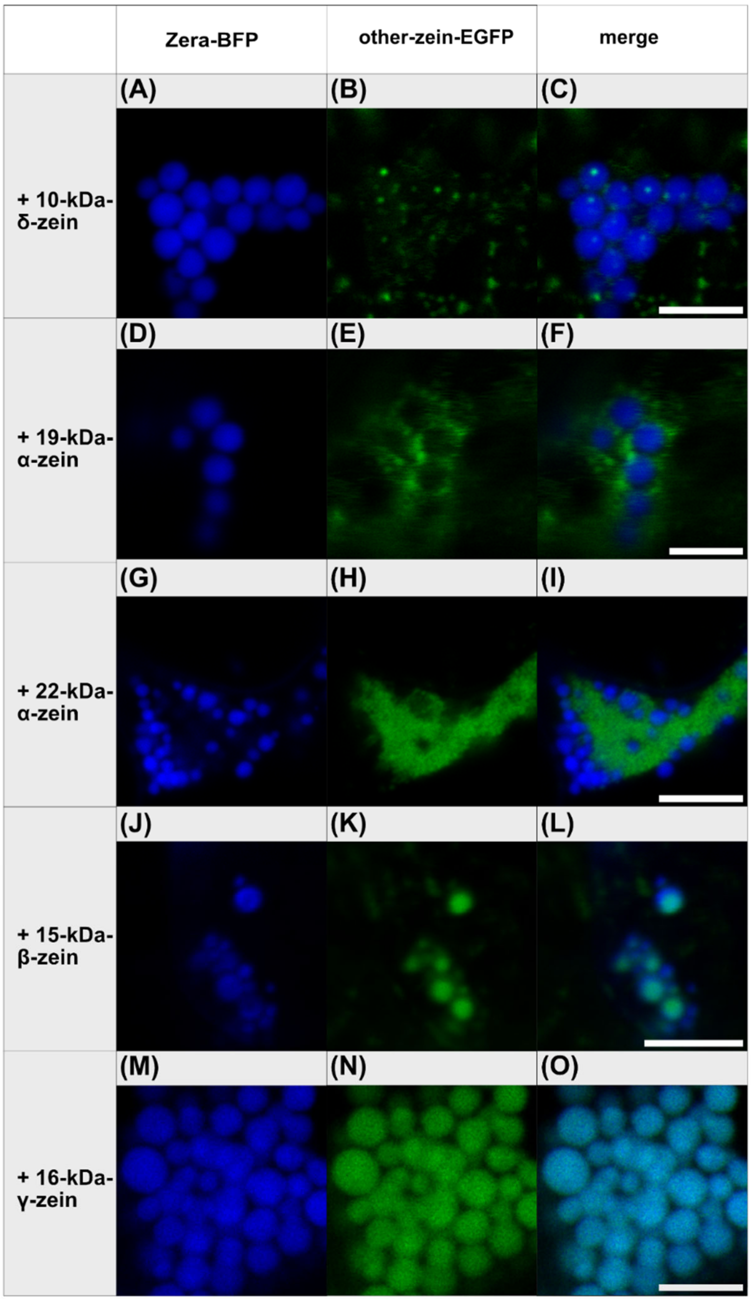

Supplementary Figure S6. Co-expression of Zera-BFP with zein-EGFP constructs (individual/single channel data for merged images shown in Figure 1). First panel: Zera-BFP; second panel: zein-EGFP-fusion of the zeins listed on the left. Third panel: overlay images. (A-C) Zera-BFP + 10-kDa-δ-zein-EGFP, (D-F) Zera-BFP + 19-kDa-α-zein-EGFP, (G-I) Zera-BFP + 22-kDa-α-zein-EGFP, (J-L) Zera-BFP + 15-kDa-β-zein-EGFP, (M-O) Zera-BFP + 16-kDa-γ-zein-EGFP. Scale bar 5 µm.

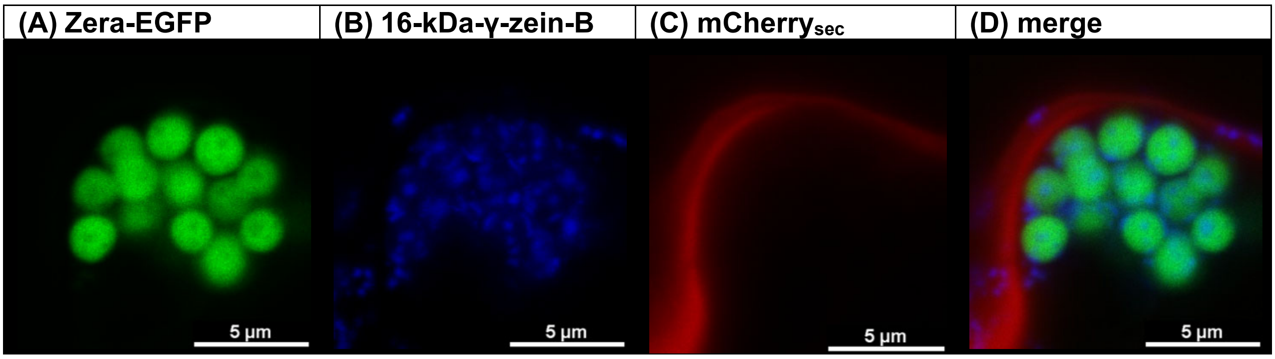

Supplementary Figure S7. Co-expression of of Zera-EGFP (A), 16-kDa-γ-zein-BFP (B), and secretory mCherry (C) (representative images recorded with confocal laser scanning microscopy). (D) overlay images. Scale bar 5 µm.

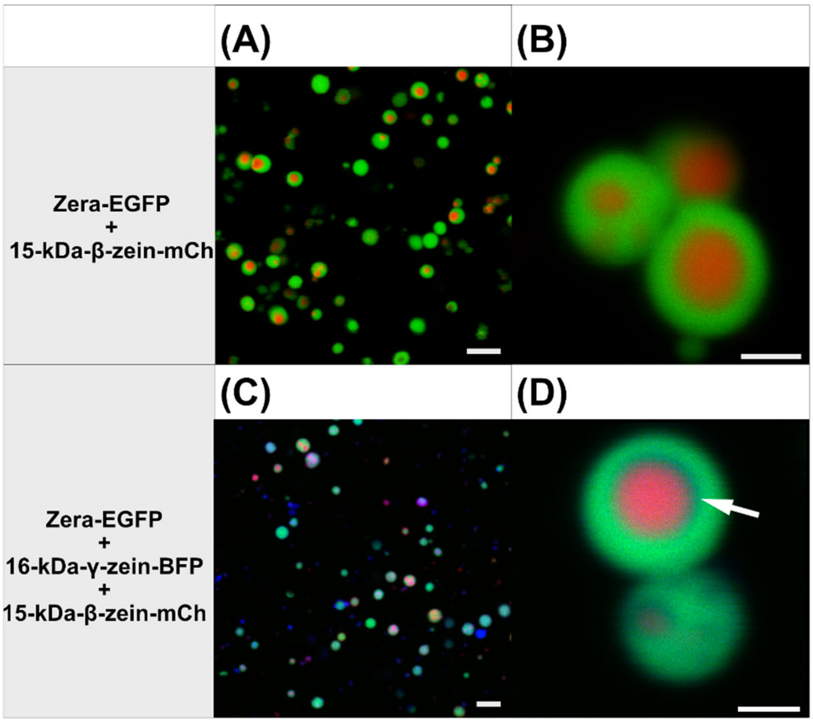

Supplementary Figure S8. Confocal laser scanning microscopy images of purified multi-layered protein bodies. (A, B) Protein bodies resulting from the co-expression of Zera-EGFP and 15-kDa-β-zein-mCherry; merged channels. (C, D) Protein bodies resulting from the co-expression of Zera-EGFP, 16-kDa-γ-zein-BFP and 15-kDa-β-zein-mCherry; merged channels. Left panel - overview of the abundance of multicolor protein bodies in the sample; scale bar 5 µm. Right panel – close-up illustrating the internal structure of individual protein bodies; scale bar 1 µm. White arrow indicates 16-kDa-γ-zein-BFP at the interface between the shell (Zera-EGFP) and the core (15-kDa-β-zein-mCherry). For easier interpretation, the blue channel was manually enhanced in (D) during image processing.

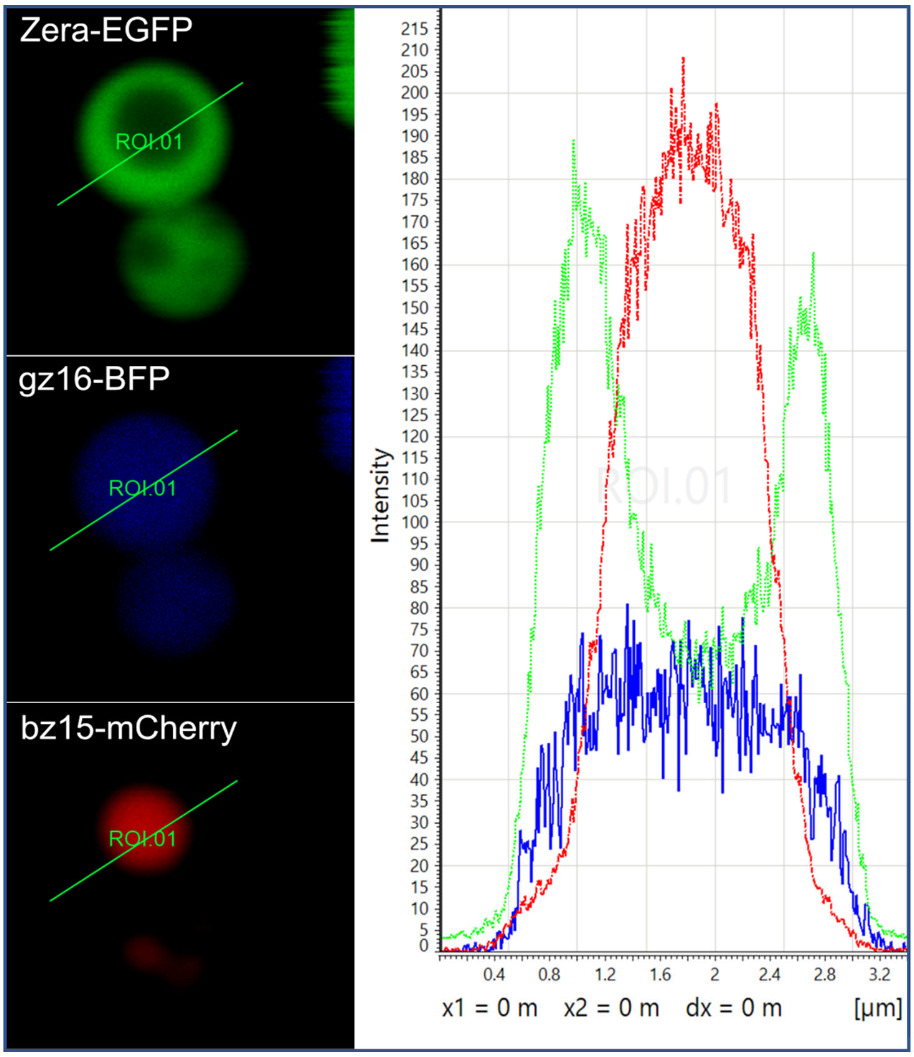

Supplementary Figure S9. Confocal laser scanning microscopy images of purified multi-layered protein bodies resulting from the co-expression of Zera-EGFP, 16-kDa-γ-zein-BFP and 15-kDa-β-zein-mCherry. Left panel: Individual (single) channel images showing the analyzed region of interest (ROI). Right panel: Intensity profiles were recorded individually for each channel (X-axis indicates the respective position on the ROI line, Y-axis indicates the intensity of the signal).
